# Smartphone use on the toilet and the risk of hemorrhoids

**DOI:** 10.1371/journal.pone.0329983

**Published:** 2025-09-03

**Authors:** Chethan Ramprasad, Colin Wu, Jocelyn Chang, Vikram Rangan, Johanna Iturrino, Sarah Ballou, Prashant Singh, Anthony Lembo, Judy Nee, Trisha Pasricha

**Affiliations:** 1 Division of Gastroenterology, Beth Israel Deaconess Medical Center, Boston, Massachusetts, United States of America; 2 Harvard Medical School, Boston, Massachusetts, United States of America; 3 Division of Gastroenterology, Department of Internal Medicine, Michigan Medicine, Ann Arbor, Michigan, Michigan, United States of America; 4 Department of Gastroenterology, Hepatology and Nutrition, Cleveland Clinic, Cleveland, Ohio, United States of America; Baotou Medical College, CHINA

## Abstract

Smartphones are ubiquitous in daily life, with many people now using them while sitting on the toilet. Despite anecdotal evidence that length of time spent on the toilet is a risk factor for hemorrhoids, a multivariate analysis of smartphone use has not been performed. This study examines the correlation between smartphone use on the toilet and prevalence of hemorrhoids. A cross-sectional study was conducted among adult patients undergoing screening colonoscopy at Beth Israel Deaconess Medical Center. Participants completed survey questions regarding their smartphone habits while using the toilet, Rome IV questionnaires, and additional behaviors including straining, fiber intake and levels of physical activity. Presence of hemorrhoids were evaluated endoscopically and independently rated by two blinded endoscopists. Categorical variables were analyzed using chi-square tests and linear variables with regression analysis. A total of 125 adult participants completed the survey and 43% had hemorrhoids visualized on colonoscopy. Participants who used smartphones on the toilet were younger than non-users (mean ages 55.4 vs. 62.1, p = 0.001). Of all respondents, 66% used smartphones while on the toilet. Participants who used smartphones on the toilet spent significantly more time there than those who did not, with 37.3% of smartphone users spending more than five minutes per visit on the toilet, compared to 7.1% of non-smartphone users (p = 0.006). Furthermore, in a multivariate logistic regression, smartphone use on the toilet was associated with a 46% increased risk of hemorrhoids (p = 0.044) after adjusting for age, sex, BMI, exercise activity, straining and fiber intake. The most common activity performed while on the toilet was reading “news” (54.3%), followed by “social media” (44.4%). The study suggests that prolonged engagement with smartphones while using the toilet may be associated with an increased prevalence of hemorrhoids.

## Introduction

Smartphone use has become integrated into daily life, influencing behaviors related to personal health and hygiene. A growing trend is the use of smartphones while sitting on the toilet to read news, to engage in social media, or simply to pass the time. This increasingly common behavior could have significant health implications, most of which have not been adequately studied.

Hemorrhoids are the third most common outpatient gastrointestinal diagnosis with nearly 4 million office and emergency department visits annually [1] and over $800 million annually in healthcare expenditure [2]. More patients seek medical care for hemorrhoids than for colon cancer, diverticular disease, irritable bowel syndrome or inflammatory bowel disease [1]. Despite this significant burden to our health systems, there is little consensus on identifiable risk factors for development of hemorrhoids.

Historically, hemorrhoids have been associated with a number of risk factors including constipation and straining with defecation [3]. Others suggest that it is prolonged sitting on the toilet, such as while reading the newspaper on the toilet [4]. Dietary factors such as low intake of dietary fiber have been linked to hemorrhoids due to harder stools and increased straining [1,5]. Pregnancy is thought to increase risk due to increased pressure in the pelvic vasculature [6]. Additionally, obesity, sedentary behavior without physical activity are thought to increase the risk of hemorrhoids [7,8].

Despite anecdotal observation, a multivariate analysis linking smartphone use with hemorrhoids has not been conducted. In this cross-sectional study, we evaluate the association between smartphone use on the toilet with risk of hemorrhoids.

## Methods

A cross-sectional survey was conducted recruiting adult patients undergoing screening colonoscopy at Beth Israel Deaconess Medical Center (BIDMC) from 01/08/2024–15/12/2024. The BIDMC Institutional Review Board approved of the study protocol (Protocol #2024P000266) and waived written informed consent for this study. Eligible participants were approached prior to their procedure and provided verbal informed consent to participate in the study, which was documented and witnessed by research staff. Data collection involved a set of structured questionnaires that were administered online, including demographic variables such as age, gender, body mass index (BMI), and lifestyle factors, such as physical activity levels using the validated International Physical Activity Questionnaire [9], and dietary habits, specifically fiber intake. Participants were also asked to report their smartphone habits while using the toilet, including the frequency and duration of use, as well as specific activities performed during this time. Lastly, participants completed the Rome IV questionnaire, which assesses gastrointestinal function and symptoms related to bowel disorders, including straining [10]. To evaluate the presence of hemorrhoids, as a binary variable, the endoscopic reports from each participant’s colonoscopy were reviewed for direct visualization findings, confirming the diagnosis. A sensitivity analysis was performed in which a subset of high-quality endoscopic images of retroflexion in the rectum from this sample were reviewed for presence of internal hemorrhoids by two independent endoscopists.

Statistical analysis was performed using R software (version 4.1.0). Categorical variables were analyzed using chi-square tests to determine differences between smartphone users and non-users, as well as other relevant demographic groups. For continuous data, linear regression analysis was employed to assess the association between smartphone usage and the outcome of interest (prevalence of hemorrhoids), while controlling for potential confounders including sex, BMI, physical activity, and fiber intake. Multivariate logistic regression was specifically utilized to calculate odds ratios for the risk of developing hemorrhoids associated with smartphone use, adjusting for all aforementioned variables. P-values of less than 0.05 were considered statistically significant. Cohen’s kappa statistic was assessed to determine agreement between the endoscopic assessment of hemorrhoids as documented during colonoscopy and determination of hemorrhoids based on subsequent review of images of retroflexion in the rectum by two independent endoscopists.

## Results

Of 143 consecutive participants who were invited, a total of 125 participants completed the survey in its entirety (87.4%) and were subsequently included in the study analysis. Of all respondents, 83 (66%) used smartphones while on the toilet. Participants who used smartphones on the toilet were younger than non-users (mean ages 55.4 vs. 62.1, p = 0.001). As measured by MET (metabolic equivalent time), smartphone users on the toilet had significantly less exercise per week than non-smartphone users (p = 0.017). There were no other differences in baseline characteristics including sex, BMI, and reported Rome IV criteria for irritable bowel syndrome or functional constipation (Table 1).

**Table 1:** Patient-reported baseline characteristics.

Characteristic	No smartphone use on toilet	Smartphone use on toilet	p-value
	N = 42	N = 83	
Age, mean (SD)	62.1 (10.3)	55.4 (9.9)	0.001
Sex, n (%)			0.701
Male	21 (50.0)	46 (55.4)	
Female	21 (50.0)	37 (44.6)	
Number of live births			0.215
0	3 (15.0)	11 (32.4)	
1	1 (5.0)	6 (17.6)	
2	8 (40.0)	11 (32.4)	
3	6 (30.0)	5 (14.7)	
> 3	2 (10.0)	1 (2.9)	
BMI, mean (SD)	28.09 (5.23)	28.55 (6.15)	0.681
Hemorrhoid(s) on colonoscopy, n (%)	16 (38.1)	42 (50.6)	0.257
Frequency of longer time sitting due to phone use^a^, n (%)			<0.001*
Never	42 (100.0)	46 (55.4)	
1–2 times per month	0 (0.0)	8 (9.6)	
1–2 times per week	0 (0.0)	24 (28.9)	
Most of the time	0 (0.0)	3 (3.6)	
All the time	0 (0.0)	2 (2.4)	
Rome IV Diagnosis, n (%)			
Functional Constipation	0 (0.0)	3 (3.6)	0.211
Functional Diarrhea	2 (4.8)	3 (3.6)	0.757
IBS-C	1 (2.4)	0 (0.0)	0.159
IBS-D	2 (4.8)	2 (2.4)	0.477
IBS-U	1 (2.4)	2 (2.4)	0.992
Other	36 (85.7)	73 (88.0)	0.726
Diverticulosis on colonoscopy, n (%)	19 (45.2)	26 (31.3)	0.182
Straining, n (%)	9 (21.4)	23 (27.7)	0.447
Physical activity (MET)^b^ score, mean (SD)	9840 (15320)	4570 (6640)	0.017*
Read material other than smartphone on toilet, n (%)	5 (11.9)	32 (38.6)	0.005*
No reading on toilet, n (%)	36 (87.8)	0 (0.0)	<0.001*
High fiber diet^c^, n (%)	29 (69.0)	32 (38.6)	0.492
Laxative use, n (%)	2 (4.8)	3 (3.6)	0.757
Elevates knees above waist on toilet^d^, n (%)	1 (2.4)	6 (7.2)	0.483
Bidet use, n (%)	5 (11.9)	10 (12.0)	1.000

^a^The question was phrased as follows: “Do you ever sit on the toilet using on your phone longer than you intended?”.

^b^MET, metabolic equivalent (minutes per week).

^c^High fiber diet was defined as being on a fiber supplement or daily reported fiber intake of 25-30g/day.

^d^The question was phrased as follows: “Do you use a stool, squatty potty, or other device to raise your knees above your waist while sitting on the toilet?”.

*Statistically significant (p < 0.05).

**Fig 1:**
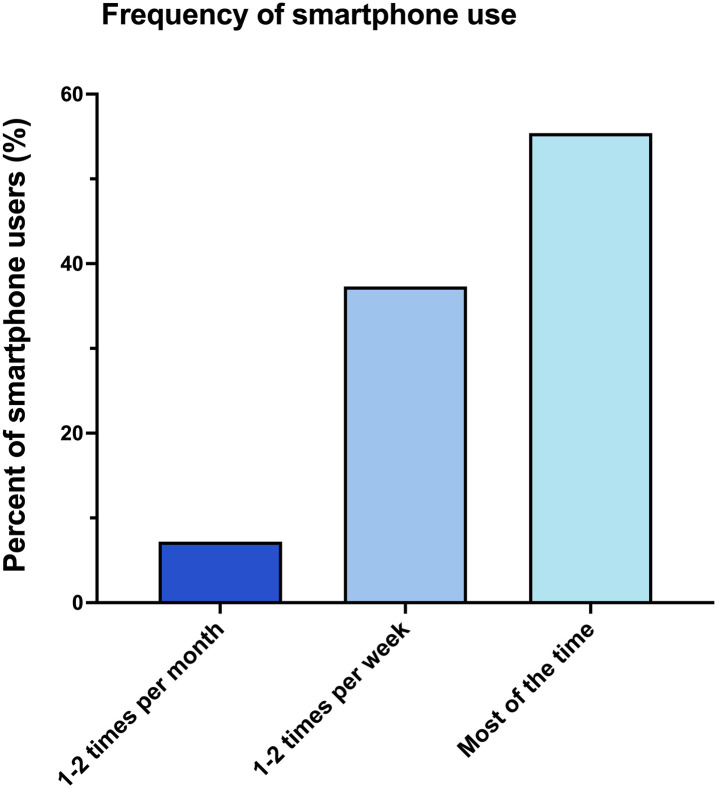
Among users, frequency of smartphone use on the toilet.

Among those who endorsed using a smartphone on the toilet, 93% endorsed using it on the toilet at least 1–2 times per week or more (Fig 1). The majority (55.4%) of smartphone users on the toilet reported using their smartphone most of the time.

**Fig 2:**
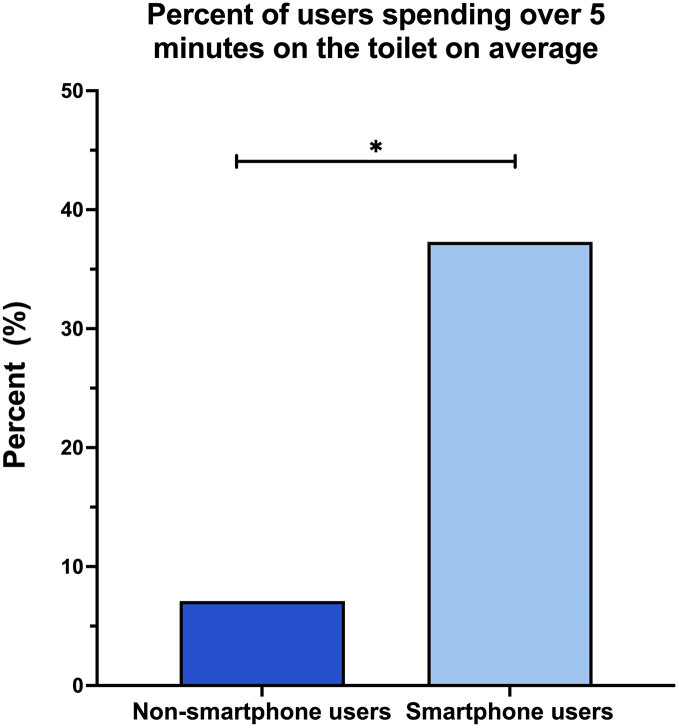
Percentage Spending Over 5 Minutes on Toilet.

Participants who used smartphones on the toilet spent significantly more time there than those who did not (Table 2), with 37.3% of smartphone users spending more than five minutes per visit on the toilet, compared to 7.1% of non-smartphone users (p = 0.006) (Fig 2). Furthermore, among smartphone users, there was a trend toward a sex difference in time spent on the toilet. Specifically, upon stratification by sex, males appeared more likely than females to spend 6 minutes or more on the toilet, though this difference did not reach statistical significance (χ² = 3.56, *p* = 0.059).

**Table 2:** Time spent on the toilet.

	No smartphone use on toilet	Smartphone use on toilet	p-value
	N = 42	N = 83	
Time on toilet, n (%)			0.006*
< 1 minute	8 (19.0)	7 (8.4)	
2–5 minutes	31 (73.8)	45 (54.2)	
6–10 minutes	3 (7.1)	20 (24.1)	
11–15 minutes	0 (0.0)	8 (9.6)	
> 15 minutes	0 (0.0)	3 (3.6)	

**Fig 3:**
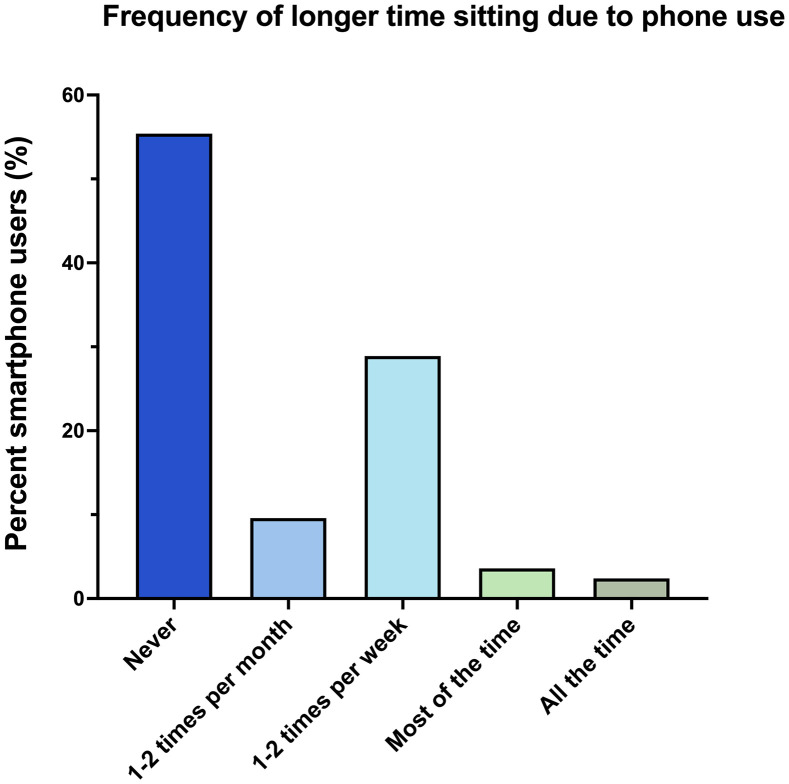
Among users, frequency of longer time sitting on the toilet due to smartphone use.

Even so, only 35% of these users acknowledged that their smartphone use resulted in more time spent on the toilet at least 1–2 times per week or more (Fig 3).

**Fig 4:**
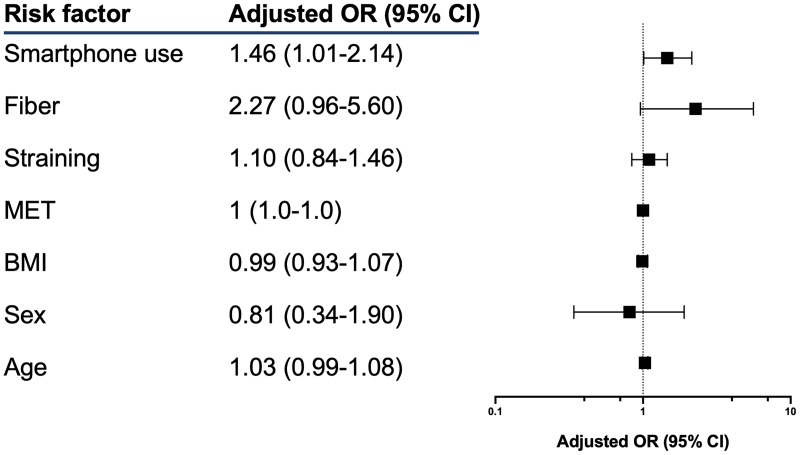
Multivariate Analysis Predicting Hemorrhoid Risk.

43% of all respondents had hemorrhoids visualized on colonoscopy. In a multivariate logistic regression, smartphone use on the toilet was associated with a 46% increased risk of hemorrhoids (p = 0.044) after adjusting for age, sex, BMI, exercise activity, straining, and fiber intake (Fig 4).

**Fig 5:**
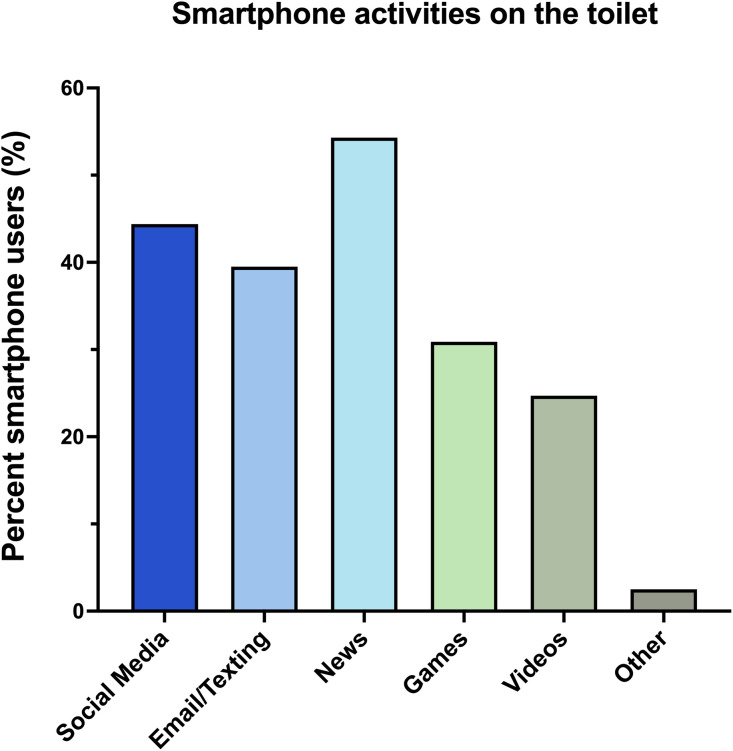
Smartphone Use Activities.

Among users of smartphones on the toilet, the most common activity performed was reading “news” (54.3%), followed by “social media” (44.4%) (Fig 5).

In a sensitivity analysis, two independent endoscopists demonstrated good interrater reliability in determining presence of internal hemorrhoids with 75% agreement on a subset of 45 high-quality endoscopic images of retroflection in the rectum. There was furthermore substantial agreement between this assessment and documentation by the endoscopist who performed the colonoscopy with a Cohen’s kappa of 0.62.

## Discussion

The findings from this study suggest that there is an increased prevalence of hemorrhoids among people who use smartphones while sitting on the toilet. Furthermore, smartphone users spent considerably more time on the toilet compared to non-smartphone users, with many spending more than five minutes on the toilet per visit.

Whereas some studies have historically linked hemorrhoids with increased straining, our results from this population do not support this hypothesis. In our study, there were no differences in straining between smartphone users and non-smartphone users, and in our multivariate model, straining was not independently predictive of hemorrhoids. Furthermore, there were no baseline differences in constipation or Rome IV diagnoses between these groups. It is possible that time spent on the toilet is a more accurate predictor of hemorrhoids than straining. This extended duration may be linked to the passive engagement that smartphones facilitate, potentially resulting in prolonged sitting and increased pressure in the hemorrhoidal cushions. While prolonged sitting outside of the toileting environment, i.e., at a desk at work or at home at leisure, this kind of sitting typically involves some support to the pelvic floor through a chair or couch. Prolonged sitting in general has not definitively been shown to be linked to hemorrhoid development [3]. However, we propose that sitting on a standard toilet seat, without any support to the pelvic floor, disproportionately increases pressure in the hemorrhoidal cushions. As this pressure persists over time, these cushions may become engorged and thereby develop into appreciable hemorrhoids. Despite an objective report of prolonged sitting among smartphone users while on the toilet, our study reveals an interesting disconnect between actual behavior and attribution of said behavior to smartphone use, reinforcing the idea that this is an inadvertent and unintended consequence.

In our study, younger age adults were more likely to use smartphones on the toilet than older adults which could demonstrate changing behaviors and their implications for health. As technology becomes increasingly common, habits such as using smartphones on the toilet may contribute to the rising incidence of conditions previously thought to be influenced primarily by dietary and lifestyle factors. Furthermore, we found that smartphone users on the toilet engaged in less exercise per week than non-smart phone users, which could signify a higher level of engagement with technology and a more sedentary lifestyle outside of the toileting environment. Previous studies have noted a correlation with low levels of physical activity and high sedentary behavior with increased smartphone use [11,12]. We hypothesize that smartphone use, which can be highly-stimulating, and steal time away from activities that may be more physically active, engenders a habit of frequent engagement that these individuals reinforce while on the toilet.

Understanding the implications of increasing smartphone use across multiple facets of our well-being has great public health value. It has been well-documented that smartphone use and social media use, particularly among youth, is correlated with increased depression, anxiety, and poor sleep [13]. Furthermore, a 2024 study analyzing over 440,000 individuals in the U.K. Biobank found an association between weekly mobile phone usage and incident cardiovascular disease risk [14]. These trends and our findings emphasize the need for healthcare providers to consider lifestyle behaviors, including technology use, when discussing health concerns with patients—and specifically inquiring about smartphone use patterns in conversations about gastrointestinal health.

This study has several limitations that must be considered. The presence of hemorrhoids amongst participants in our study was determined based on endoscopist colonoscopy reports which can be inherently subjective as some endoscopists may not document hemorrhoids, may not perform retroflexion, or may vary in their ability to identify them. To address this limitation, our sensitivity analysis demonstrated good interrater reliability between two independent endoscopists reviewing high-quality images as well as substantial agreement between their assessment and that of the endoscopist performing the colonoscopy. Additional limitations include the cross-sectional design that restricts the ability to establish causation, and the reliance on self-reported data, particularly of straining and time on toilet, that introduces potential recall bias. Furthermore, the study population, composed of adults undergoing screening colonoscopy age 45 and older, may not be representative of the general population. Lastly, this study did not assess how long participants have been using smartphones on the toilet. Presumably, more years of smartphone use could lead to worsening toilet habits and increased risk of hemorrhoids.

Despite the study’s limitations, we believe these findings contribute information that may be valuable in routine clinical care, namely in bolstering advice to restrict smartphone use while on the toilet to under 5 minutes. Additionally, our findings suggest a potential behavioral difference in time spent on the toilet by sex that we were likely under-powered to fully determine. This may warrant further investigation in larger cohorts, including differences in related toileting behaviors including straining.

While it is a general adage that one should avoid prolonged toilet sitting, our study adds a concrete risk association between hemorrhoids and smartphone use, which may inadvertently prolong intended time on the toilet. Future studies could benefit from longitudinal designs that track changes in smartphone usage and hemorrhoid incidence over time. Additionally, qualitative research exploring user experiences and perceptions of smartphone use on the toilet could provide a deeper understanding of behavioral motivations and attitudes. Lastly, the implementation of educational interventions aimed at promoting healthier toilet habits, including limiting prolonged sitting during smartphone use such as with timers, may represent a promising avenue for reducing the prevalence of hemorrhoids in the population.

## References

[1] Peery AF, Crockett SD, Barritt AS, Dellon ES, Eluri S, Gangarosa LM, et al. Burden of Gastrointestinal, Liver, and Pancreatic Diseases in the United States. Gastroenterology. 2015;149(7):1731–41.e3. doi: 10.1053/j.gastro.2015.08.045 26327134 PMC4663148

[2] Yang JY, Peery AF, Lund JL, Pate V, Sandler RS. Burden and Cost of Outpatient Hemorrhoids in the United States Employer-Insured Population, 2014. Am J Gastroenterol. 2019;114(5):798–803. doi: 10.14309/ajg.0000000000000143 30741736 PMC6502684

[3] Peery AF, Sandler RS, Galanko JA, Bresalier RS, Figueiredo JC, Ahnen DJ, et al. Risk Factors for Hemorrhoids on Screening Colonoscopy. PLoS One. 2015;10(9):e0139100. doi: 10.1371/journal.pone.0139100 26406337 PMC4583402

[4] Dehn TC, Kettlewell MG. Haemorrhoids and defaecatory habits. Lancet. 1989;1(8628):54–5. doi: 10.1016/s0140-6736(89)91717-0 2563041

[5] Lokarjana L, Kanseria T, Roslaeni R, Pratama AY. The relationship between low fiber consumption and the incidence of haemorrhoids patients. In: 12th Annual Scientific Meeting, Medical Faculty, Universitas Jenderal Achmad Yani, International Symposium on “Emergency Preparedness and Disaster Response during COVID 19 Pandemic” (ASMC 2021). Atlantis Press; 2021 Jul 24, p. 197–9.Lokarjana L, Kanseria T, Roslaeni R, Pratama AY. The relationship between low fiber consumption and the incidence of haemorrhoids patients. In: 12th Annual Scientific Meeting, Medical Faculty, Universitas Jenderal Achmad Yani, International Symposium on “Emergency Preparedness and Disaster Response during COVID 19 Pandemic” (ASMC 2021), 2021, p. 197–9.

[6] Hong YS, Jung KU, Rampal S, Zhao D, Guallar E, Ryu S, et al. Risk factors for hemorrhoidal disease among healthy young and middle-aged Korean adults. Sci Rep. 2022;12(1):129. doi: 10.1038/s41598-021-03838-z 34996957 PMC8741788

[7] Malviya VK, Diwan S, Sainia TK, Apte A. Demographic study of hemorrhoid with analysis of risk factors. Surgical Update: Int J surg Orthopaedics. 2019;5(1):7–13.

[8] Kibret AA, Oumer M, Moges AM. Prevalence and associated factors of hemorrhoids among adult patients visiting the surgical outpatient department in the University of Gondar Comprehensive Specialized Hospital, Northwest Ethiopia. PLoS One. 2021;16(4):e0249736. doi: 10.1371/journal.pone.0249736 33878128 PMC8057569

[9] Craig CL, Marshall AL, Sjöström M, Bauman AE, Booth ML, Ainsworth BE, et al. International physical activity questionnaire: 12-country reliability and validity. Med Sci Sports Exerc. 2003;35(8):1381–95. doi: 10.1249/01.MSS.0000078924.61453.FB 12900694

[10] Mearin F, Lacy BE, Chang L, et al. Bowel Disorders. Gastroenterology. 2016;150(6):1342–63. 10.1053/j.gastro.2016.02.031 27144627

[11] Grimaldi-Puyana M, Fernández-Batanero JM, Fennell C, Sañudo B. Associations of Objectively-Assessed Smartphone Use with Physical Activity, Sedentary Behavior, Mood, and Sleep Quality in Young Adults: A Cross-Sectional Study. Int J Environ Res Public Health. 2020;17(10):3499. doi: 10.3390/ijerph17103499 32429550 PMC7277080

[12] Shimoga SV, Erlyana E, Rebello V. Associations of Social Media Use With Physical Activity and Sleep Adequacy Among Adolescents: Cross-Sectional Survey. J Med Internet Res. 2019;21(6):e14290. doi: 10.2196/14290 31215512 PMC6604510

[13] Yang J, Fu X, Liao X, Li Y. Association of problematic smartphone use with poor sleep quality, depression, and anxiety: A systematic review and meta-analysis. Psychiatry Res. 2020;284:112686. doi: 10.1016/j.psychres.2019.112686 31757638

[14] Zhang Y, Ye Z, Zhang Y, Yang S, Liu M, Wu Q, et al. Regular Mobile Phone Use and Incident Cardiovascular Diseases: Mediating Effects of Sleep Patterns, Psychological Distress, and Neuroticism. Can J Cardiol. 2024;40(11):2156–65. doi: 10.1016/j.cjca.2024.06.006 39230550

